# Modification of the Sensory Profile and Volatile Aroma Compounds of Tomato Fruits by the Scion × Rootstock Interactive Effect

**DOI:** 10.3389/fpls.2020.616431

**Published:** 2021-01-20

**Authors:** Maja Jukić Špika, Gvozden Dumičić, Karolina Brkić Bubola, Barbara Soldo, Smiljana Goreta Ban, Gabriela Vuletin Selak, Ivica Ljubenkov, Marija Mandušić, Katja Žanić

**Affiliations:** ^1^Department of Applied Sciences, Institute for Adriatic Crops and Karst Reclamation, Split, Croatia; ^2^Centre of Excellence for Biodiversity and Molecular Plant Breeding, Zagreb, Croatia; ^3^Department of Plant Sciences, Institute for Adriatic Crops and Karst Reclamation, Split, Croatia; ^4^Department of Agriculture and Nutrition, Institute of Agriculture and Tourism, Poreč, Croatia; ^5^Department of Chemistry, Faculty of Science, University of Split, Split, Croatia

**Keywords:** *Solanum lycopersicum* (L.), grafting, firmness, juiciness, hydroponic, sensory quality, volatile aroma compound, texture

## Abstract

Sensory quality is of increasing importance to consumer decisions in choosing a product, and it is certainly an important factor in repurchasing in terms of meeting the necessary aroma quality and taste properties. To better understand the effects of rootstocks and scions on fruit quality, the sensory profile and volatile aroma composition of the fruits of hydroponically grown tomato plants were evaluated. Experiments were established using the tomato cultivars Clarabella and Estatio as scions during two spring-summer seasons. In both experiments, the scion plants were self-grafted or grafted onto rootstocks of cultivars Arnold, Buffon, Emperador, and Maxifort, with the exception that in experiment 1, the Estatio scion was not grafted onto Buffon. The scions and rootstocks caused differences in observed sensory properties in both experiments. For most of the sensory traits, interaction effects between scion and rootstock were observed. Compared to those obtained from self-grafted Clarabella, the fruits obtained from Clarabella grafted onto Buffon in the first experiment and Clarabella grafted onto Arnold in the second experiment were sweeter by one measurement unit. The contents of seven aldehydes, six alcohols, five terpenes and two ketones were determined. A lower accumulation of total aldehydes, 22–45%, due to lower amounts of pentanal, (*E*)-2-heptanal and (*E,E*)-2,4-decadienal, was found in the fruits from plants where Estatio was rootstock compared with the other rootstocks treatments. Clarabella as a rootstock increased (*Z*)-3-hexenal + (*E*)-2-hexenal accumulation from 35 to 65%. Grafting Clarabella onto the tested rootstocks led to a change in the composition of volatile compounds, while differences between the combinations with Estatio as a scion were generally not recorded. Fruits from self-grafted Clarabella had higher (*Z*)-3-hexenal + (*E*)-2-hexenal concentrations than did fruits from Clarabella grafted onto Arnold (for 54%) and Emperador (for 68%), and in the second experiment, grafting onto all commercial rootstocks reduced (*Z*)-3-hexenal + (*E*)-2-hexenal concentrations, from 25 to 74%, compared to those from self-grafted Clarabella. Higher (+)-2-carene and (−)-caryophyllene oxide concentrations were attained in plants in which Clarabella was grafted onto Maxifort (by 56%) and plants in which Estatio was grafted onto Arnold (by 36%) compared to self-grafted plants. This study showed the possibility of altering the composition of volatile aroma compounds and sensory properties of tomato fruits by the use of grafting techniques.

## Introduction

To combat the consequences of climate change and achieve profitability, tomato production techniques undergo continual improvements. Good agronomic performance, such as mitigation of stress effects, fruit size uniformity, color appearance, and high yield, is an important factor in tomato breeding programs (Paolo et al., 2018). Thus, vegetable grafting has gained a great deal of attention for overcoming problems, not only to overcome soil-borne diseases or airborne pests but also to improve crop performance under unfavorable conditions, such as water, salt, nutrient, and temperature stresses (Goreta et al., 2008; Rouphael et al., 2008; Colla et al., 2010; King et al., 2010; Lee et al., 2010; Schwarz et al., 2010; Ban et al., 2014). Moreover, many rehearses reported grafting as a tool to overcome multiple abiotic stresses which usually occur under open field conditions (Khah et al., 2006; Rouphael et al., 2008; Djidonou et al., 2016; Casals et al., 2018). Compared with non-grafted plants, commercially available rootstocks usually have a larger and stronger root system that enables them to absorb water and nutrients much more effectively (Lee et al., 2010). These rootstock characteristics have led to a noticeable increase in fruit yield – one of the major advantages of using grafted vegetable transplants (Lee et al., 2010; Schwarz et al., 2010). However, because agronomic performance has been the focus in most cases, organoleptic characteristics have been neglected (Klee and Tieman, 2013; Paolo et al., 2018), and finding a scion/rootstocks combination that improves fruit quality and reconciled without affecting sensory quality is still a challenge.

The flavor is a combined sensation of taste and aroma, and sugars, acids and volatile compounds are their major determinants (Yilmaz, 2001b; Bennett, 2012; Tieman et al., 2012). A plethora of volatile compounds have been identified, and the volatile profile of tomato has been investigated in depth in numerous studies (Buttery and Ling, 1993; Krumbein and Auerswald, 1998; Maul et al., 1998; Auerswald et al., 1999a; Krumbein et al., 2004; Marković et al., 2007; Viljanen et al., 2011; Oluk et al., 2019). Identified tomato volatiles are metabolically derived from fatty acids, aliphatic amino acids, phenolic compounds and terpenoids such as β-carotene and lycopene (Bertin and Génard, 2018).

Linking chemically assessed volatile compounds with tomato flavor by sensory analysis has been a subject for several studies, while consumer preference studies highlight fruit texture (excluding overall flavor) as an influencing trait for purchasing decisions (Causse et al., 2010). Indeed, an integrated perception of odor and taste is distinctly linked to texture by the mechanism of tissue disruption and the mode of cellular component release, thus influencing juiciness and the overall fruit flavor (Auerswald et al., 1999a; Baldwin et al., 2000; Arefi et al., 2015). Quantitative descriptive analysis can provide a set of attributes describing external appearance and texture-related properties such as skin thickness, pulp firmness, mealiness and juiciness. Additionally, it can be used to integrate the various aromas of food, such as that perceived by smelling through the front of the nose and the retro-nasal cavity and the aroma that is released during chewing.

Grafting has not been primarily used as a method for fruit quality improvement, and in some previous investigations, yield and quality have usually been contradictory traits (Klee and Tieman, 2013; Kyriacou et al., 2017). Little information is available about the sensory property changes influenced by grafting. Di Gioia et al. (2010) found no changes in sensory attributes as influenced by different rootstocks (Mauro et al., 2020), and significant genotype × grafting interactions (Casals et al., 2018) were noticed in studies in which trained panels were used. In other experiments, a negative effect of grafting was observed, manifested as a reduction in sweetness, acidity, and flavor attribute intensities. Sweetness is the most frequently examined single quality trait, and contrasting results were found not only in terms of the scion used but also the rootstock used (Turhan et al., 2011; Schwarz et al., 2013; Gajc-Wolska et al., 2015; Riga, 2015). This suggests that, on the basis of the different rootstock/scion combinations, sensory properties can be modified in accordance with the market requirements. Importantly, other non-specific sweetness parameters, such as acidity and aroma volatiles, influence perceived sweet sensation (Tieman et al., 2012).

Several review articles (Rouphael et al., 2010; Fallik and Ilic, 2015; Singh et al., 2017) covered all resent findings of grafting effect on tomato fruit quality, including volatile aroma compounds. However, the interactive effect of the scion × rootstock affecting volatile aroma compounds has not been sufficiently investigate and explained. Krumbein and Schwarz (2013) reported a significant effect of grafting on two-thirds of the volatiles detected. Three volatiles (methyl salicylate, guaiacol, and eugenol) increased in abundance in response to grafting, whereas the concentrations of benzaldehyde, β-ionone and geranylacetone decreased. In addition, the scions studied interacted with the rootstocks, leading to different changes in the composition of identified volatile compounds. Similarly, grafting tomato onto three rootstocks commonly used in Mediterranean greenhouse production (Mauro et al., 2020) significantly changed the volatile composition; specifically, each rootstock promoted increases in a different volatile compound (Mauro et al., 2020). We consider that volatile profile variations in relation to the rootstock, scion and their interactions might be key factors in view of developing strategies of fruit quality management.

The aim of this study was to investigate the rootstock and scion effects on the sensory profile and aroma volatile compound composition of fruits of hydroponically grown tomato plants. The emphasis was on their changes induced by scion x rootstock interaction effects, aiming to preserve or enhance key sensory traits as major interests of consumers. Compared to previous studies, the experiment was established using the two tomato scions differed in type of fruit, Clarabella (beef) and Estatio (cluster), during two spring-summer seasons. The scion plants were self-grafted or grafted onto cultivars Arnold, Buffon, Emperador, and Maxifort, which were used as rootstocks.

## Materials and Methods

### Plant Material and the Design of the Experiments

Two experiments with grafted tomato plants were established at the Institute for Adriatic Crops at Split (43°30′17.17′′N, 16°29′49.71′′E) in the spring-summer season. The cultivars Clarabella (Rijk Zwan, Netherlands) and Estatio (Syngenta Seeds, Switzerland) were used as scions in both experiments. In experiment 1, conducted in 2016, Clarabella and Estatio scions were self-grafted or grafted onto different commercial rootstock cultivars: Arnold (Syngenta Seeds, Switzerland), Emperador (Rijk Zwan) and Maxifort (Seminis, Bayer group, Germany), while Clarabella was also grafted onto Buffon (Syngenta Seeds) rootstock. In experiment 2, conducted in 2017, both of the above mentioned scions were self-grafted or grafted onto the same four rootstocks that were used in experiment 1.

To prepare the tomato plants for both experiments, the seeds of the scions and rootstocks were sown in a heated experimental glasshouse with five separate chambers (50 m^2^ each, Schwarzmann, Slovenia) from 08 to 10 February. The seeds were sown in an organic substrate (Brill Substrate Tip 4 GmbH & Co., KG, Germany) using polystyrene plates with 228 sowing places (volume: 20 mL each) and were watered with tap water. During seedling development, the temperature ranged from 22 to 28°C. The seedlings were grafted on 02 March in experiment 1 and on 03 March in experiment 2, via splice grafting, as described by Lee et al. (2010). The grafted seedlings were cultured until callus formation, and after acclimation, they were transplanted, grown in rockwool cubes, and fertigated (in both experiments), as described by Žanić et al. (2018).

Young plants growing in rockwool cubes with five developed leaves were transplanted into rockwool slabs (7.5 cm × 20 cm × 100 cm; Kran-izol s.r.o., Czechia) on 08 April in experiment 1 and on 07 April in experiment 2. The experiments were set up in an unheated experimental glasshouse with a total area of 1,200 m^2^ (Salco, Italy). In both cases, the experimental design was a randomized block experimental design with four replicates. In both experiments, each treatment included 24 plants. Tomato transplants were arranged in one-row system, 140 cm × 50 cm, for a total of 1.43 plants/m^2^. Each plant was grown with two shoots. The plants were fertigated with a tomato nutrient solution prepared according to the methods of Sonneveld and Straver (1994) 3–14 times per day, at doses of 0.6–4.2 L per plant. The amount of nutrient solution (EC 3 dS/m and pH 5.5 – 6.5) was estimated based on the collected drainage from the previous day, which was 20–30% of the applied amount of nutrient solution. Fertigation was performed from 7 a.m. to 7 p.m., and was more frequent from 11 a.m. to 5 p.m. Each added portion included 200 mL of nutrient solution per plant. In first experiment pollination was performed by shaking the plants five time per day while the pollinators, bumblebees (Biobest Group NV, Belgium) were used in experiment 2. To conduct chemical and sensory analyses, fully colored tomato fruits without visible symptoms of physiological or mechanical damage were sampled for each combination (rootstock/scion) from the same cluster and same position (third and fourth cluster) on 27 June in experiment 1 and 04 July in experiment 2. The values of temperature parameters (average, minimal, and maximal temperature) and number of cloudy days registered for the period under observation in year 2016 and 2017 (Supplementary Figure 1) and were obtained from Meteorological and Hydrological Service of Croatia.

### Quantitative Descriptive Sensory Analyses of Tomato Fruits

Quantitative descriptive analysis (QDA) of tomato fruits was performed by nine assessors who had extensive experience in sensory analysis of different kinds of foods and who were previously trained for tomato descriptors according to the methods proposed by Auerswald et al. (1999b) and Gajewski et al. (2014). Odor, internal appearance, texture/mouthfeel, and taste/flavor of the tomato fruit were determined according to the methods proposed by Gajewski et al. (2014), using a modified profile sheet expanded with descriptors for external appearance and firmness by touch, as proposed by Auerswald et al. (1999b). Single sensory attributes were quantified using a 10 cm unstructured ordinal intensity rating scale from 0 (no perception) to 10 (the highest intensity). For overall quality evaluation, tomato fruits were graded by points from 0 (the lowest quality) to 10 (the highest quality). The samples were served in two replicates per treatment in plastic plates coded randomly. Sensory analysis was divided into two sessions with an equal number of samples. QDA was performed in a sensory laboratory under temperature- and light-controlled conditions. Each assessor worked in a separate booth, and between each sample, he or she consumed apples, bread and water (as mouthwash). An arithmetic mean of 9 scores (one per assessor) for each sensory attribute was used for subsequent data treatment. The results represent the mean of two mean values per sample obtained at two different sessions.

### Analyses of Tomato Fruit Volatile Compounds

After harvest, the tomato samples used in the study were kept at −80°C until analysis. Prior to the measurements, defrosted tomato fruits (12 g) were homogenized by a Polytron PT 1600 E (Kinematica, Luzern, Switzerland) twice for 30 s at 15,000 rpm, with a 1 min pause between cycles. The homogenized tissue (4 g) was weighed in 20 mL tubes (Supleco, Inc., Bellefonte, PA, United States), after which a small magnetic stir bar was added, and each tube was closed with silicon septa (PTFE silicone, 20 mm; Alum, United States).

Volatile compounds were extracted from the tomato fruits according to a method previously described by Marković et al. (2007), with a slight modification. Headspace solid-phase microextraction (HS-SPME) was carried out by using 2 cm long divinylbenzene/caboxen/polymethiloxane-coated fiber; the thickness of the absorbent polymer was 50/30 μm (Supleco SU 57348U, Supleco, Inc., Bellefonte). Prior to the extraction, the sample was mixed for 25 min in a water bath at 40°C such that the volatile compounds in the headspace reached equilibrium. The volatile compounds were sampled by inserting an SPME needle through the silicon septa for 20 min at 40°C.

Analysis of the volatile compounds in the tomato samples was performed using a Varian GC 3900 (Varian Inc., Palo Alto, CA, United States) gas chromatography system equipped with a split/splitless injector, flame ionization detector (FID) and CP-WAX 57 CB quartz capillary column (length 50 m, inner radius 0.25 mm and 0.2 μm film thickness; Varian Inc., Palo Alto, CA, United States). In this study, the conditions for the GC analysis were as follows: helium was used as the carrier gas at a flow rate of 5.0 mL min^–1^ and pressure of 22.6 psi (155821.5 Pa; 1 psi = 6894.76 Pa). The injection was carried out according to the splitless technique, and temperature desorption of the SPME needle was performed for 10 min at 250°C. Between two samplings, the SPME needle was cleaned by heating in the injector for 10 min at 250°C.

The starting temperature of the instrument oven was maintained at 40°C for 4 min, after which the temperature was raised in intervals of 5°C/min to 190°C, and this temperature was kept constant for 11 min. The temperature was then raised by applying the same temperature increment scheme up to 200°C, and this temperature was maintained for 8 min. The temperature of the flame ionization detector was maintained at 250°C.

The volatile compounds in the tomato fruit samples were identified by comparison with the retention time of standards. The volatile compound standards used were ≥97% pure. Given that we found the presence of n-amyl alcohol in our samples, calibration was carried out using the external reference method. The results were expressed as the mass ratio of n-amyl alcohol, in micrograms of n-amyl alcohol per kilogram (Sigma-Aldrich, St. Louis, MO, United States) (Wang et al., 2001; Marković et al., 2007). The standard mass ratio range (0.0625–0.5 mg/kg), with a correlation coefficient of 0.9620, was covered by the calibration curve. The obtained data were analyzed using GC Workstation 6.41 software (Varian Inc.) and the data from three independent measurements were collected.

### Data Analyses

By the use of proc glm of SAS software (SAS Institute Inc., Cary, NC, United States), the obtained data from this study were tested for normality and homogeneity of variance and transformed when necessary. They were then subjected to two-way analysis of variance, and when *F*-tests were significant, the means of the main factors (scion and rootstock) and their interactions were compared using Tukey’s honestly significant difference test at *P* ≤ 0.05.

## Results

### Sensory Quality

#### Experiment 1

The results of analyses of evaluated tomato fruit sensory attributes and overall quality scores in experiment 1 are presented in Table 1 and Supplementary Table 1. The results of sensory descriptive analysis included the external and internal appearance of the fruit, the flavor properties of the sliced fruit, the impression during chewing, the taste and aftertaste, and the overall fruit quality. Foreign odors and foreign tastes were not detected in any of the evaluated samples.

**TABLE 1:** Effects of scions and rootstocks on the tomato fruit sensory attribute intensities in the experiment 1.

Factor	Sensory attributes and scores															
	Fruit appearance				Odor of the cut fruit			Mouthfeel/texture				Taste and after-taste				Overall score
	shape	firm	fr col	fl col	tom	green	fruity	thick	firm	mea	juic	tom	sour	sweet	bitter	
**Scion (S)**																
Clarabella	7.26b	7.36a	7.50	5.95	7.58a	3.69	1.14b	7.28	6.36a	2.89a	7.08	7.46a	3.63b	2.24a	1.02	7.79a
Estatio	8.09a	6.16b	7.35	6.38	6.98b	3.55	1.48a	7.00	5.72b	2.43b	7.26	6.96b	4.13a	1.70b	0.93	7.16b
*F-value*	17.62	76.07	2.05	1.23	35.08	4.42	6.43	0.91	18.48	38.68	0.12	21.86	10.50	35.47	0.03	25.82
*p*	0.003	<0.0001	0.191	0.299	0.000	0.069	0.035	0.369	0.003	0.000	0.734	0.002	0.012	0.0003	0.872	0.001
**Rootstock (R)**																
Clarabella	6.48b	7.40	7.19	5.47	7.40	3.53	0.99	8.00a	7.10a	3.01a	6.40	7.26	4.29ab	1.74b	1.14	7.20bc
Arnold	7.76a	6.87	7.38	6.02	7.29	3.59	1.31	7.21b	5.89bc	2.79ab	7.12	7.14	3.98abc	2.03b	0.93	7.42bc
Buffon	7.73a	7.07	7.44	5.77	7.67	3.72	1.18	6.83b	6.03bc	2.69bc	7.48	7.66	3.35d	2.60a	1.42	8.39a
Emperador	7.73a	7.01	7.59	6.51	7.24	3.63	1.26	7.11b	6.22b	2.62bc	7.20	7.16	3.74bcd	2.00b	0.86	7.50b
Maxifort	7.67a	6.51	7.42	6.36	7.27	3.46	1.33	6.98b	5.72c	2.47c	7.24	7.25	3.59cd	1.94b	0.80	7.53b
Estatio	8.01a	6.17	7.27	6.24	7.09	3.81	1.58	6.93b	5.84bc	2.65bc	7.43	7.07	4.39a	1.69b	1.07	7.06c
*F*-value	3.51	2.21	0.89	1.74	0.66	2.40	0.47	3.78	8.36	3.17	3.24	1.13	3.93	5.58	0.84	7.25
*p*	0.050	0.153	0.528	0.231	0.661	0.130	0.787	0.047	0.005	0.049	0.068	0.420	0.043	0.017	0,559	0.008
**S × R**																
*F*-value	0.42	1.86	2.89	0.36	3.01	1.64	0.55	3.43	16.00	3.23	0.61	2.89	0.04	4.80	0.36	5.32
*p*	0.671	0.217	0.114	0.711	0.106	0.254	0.596	0.084	0.002	0.094	0.057	0.114	0.957	0.043	0.706	0.034

*The means marked with different lowercase letters in row (for each factor) are significantly different (Tukey’s test, p ≤ 0.05). Identification of sensory attributes and scores: fruit appearance, external and internal appearance: shape, fruit shape; firm, fruit firmness by touch; fr col, color of the fruit; fl col, flesh color intensity; odor of the cut fruit: tom, tomato–like odor; green, green/grass odor; mouthfeel/texture attributes: thick, skin thickness; firm, flesh firmness; mea, flesh mealiness; juic, flesh juiciness; taste and after-taste: tom, tomato flavor; overall score, overall quality score.*

The sensory properties indicated that *fruit shape* and *fruit firmness* significantly differed between scions, with Estatio scion yielding fruits with a rounded shape but with relatively low *fruit firmness* (Table 1). The Clarabella scion yield fruits with a more intense tomato-like odor, while fruits from Estatio had a higher intensity of *fruity* odor characterized by fruit ripeness notes.

Scions also differed in fruit sensory parameters evaluated during chewing (Table 1). The fruits of Clarabella were characterized by firmer and mealier flesh. Higher intensities of *tomato*-like and *sweet* sensory characteristics were detected in Clarabella fruits compared with Estatio scion fruits, which were sourer.

Rootstock influenced the intensity of sensory properties related to external appearance, mouthfeel/texture, taste attributes and overall quality score (Table 1). Clarabella as a rootstock yielded fruits with thicker skin and firmer flesh compared to those from other rootstocks. The fruits from Buffon rootstock were sweeter and were generally evaluated as having the highest sensory score.

**FIGURE 1:**
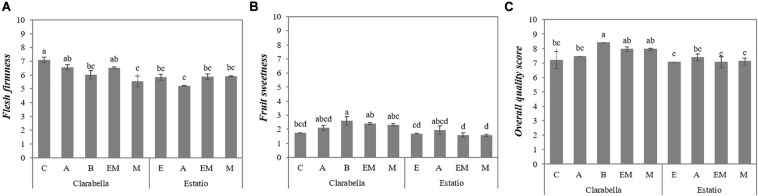
Scion × rootstock interaction effects on tomato fruit sensory attribute intensities in the experiment 1. The results are expressed as the mean values ± SEs. The different lowercase letters indicate significant differences according to the Tukey test at *p* ≤ 0.05. Clarabella and Estatio are scions that were self-grafted or grafted onto Arnold (A), Emperador (EM), or Maxifort (M) rootstocks, while only Clarabella scions were grafted onto Buffon (B) rootstocks. Identification: **(A)** Flash firmness; **(B)** Fruit sweetness; **(C)** Overall quality score.

The scion × rootstock interaction influenced the intensities of *flesh firmness* and *sweet* taste, as well as the overall quality score (Table 1 and Figure 1). *Flesh firmness* was higher for fruits from self-grafted Clarabella than for fruits from Clarabella grafted onto Buffon and Maxifort and fruits from all combinations with Estatio (Figure 1A).

Fruits from Clarabella grafted onto Buffon were sweeter than fruits from self-grafted Clarabella, while no difference in *sweet* taste was found among combinations with Estatio as the scion (Figure 1B).

Based on the information in Figure 1C, the fruits of Clarabella grafted onto Buffon had a higher overall quality score than did those of the other assessed combinations, with the exception of fruits of Clarabella grafted onto Emperador and Maxifort.

#### Experiment 2

The tomato attributes obtained via quantitative descriptive sensory analysis in experiment 2 are presented in Table 2 and Supplementary Table 2. The scion significantly influenced most of the evaluated sensory characteristics (10 out of 15). Negative sensory properties (foreign odor and taste) were not detected in any of the analyzed samples.

**TABLE 2:** Effects of scions and rootstocks on the tomato fruit sensory attribute intensities in the experiment 2.

Factor	Sensory attributes and scores															
	Fruit appearance				Odor of the cut fruit			Mouthfeel/texture				Taste and after-taste				Overall score
	shape	firm	fr col	fl col	tom	green	fruity	thick	firm	mea	juic	tom	sour	sweet	bitter	
**Scion (S)**																
Clarabella	7.10a	7.12	7.25a	6.98a	7.19a	3.19b	1.20a	5.81	5.07	3.86a	6.89	6.59a	3.27	1.66a	0.10	7.97a
Estatio	6.54b	7.18	6.62b	6.37b	5.47b	3.59a	0.47b	5.91	6.13	3.21b	6.78	5.17b	3.13	0.71b	0.11	6.34b
*F*-value	5.85	0.56	16.85	25.72	110.18	15.17	71.54	7.74	2.51	7.15	3.77	62.72	0.78	313.13	0.05	74.62
*p*	0.039	0.472	0.003	0.0007	<0.0001	0.004	<0.0001	0.213	0.148	0.025	0.084	<0.0001	0.401	<0.0001	0.821	<0.0001
**Rootstock (R)**																
Clarabella	7.23	7.32	7.26	6.79	7.08	3.71ab	1.17	6.50a	5.16	4.51	6.29	6.41	3.18	1.43a	0.10	7.56
Arnold	6.79	7.22	6.80	6.56	6.11	3.46abc	0.80	5.91c	5.24	3.39	6.78	5.79	3.05	1.43a	0.09	7.05
Buffon	6.82	6.96	7.07	6.89	6.33	3.12c	0.87	5.61de	5.15	3.54	7.03	5.95	3.08	1.23ab	0.10	7.33
Emperador	6.81	7.29	6.89	6.59	6.40	3.32bc	0.79	5.83cd	5.34	3.65	6.87	5.91	3.31	1.09b	0.08	7.29
Maxifort	6.86	6.94	6.96	6.63	6.45	3.23c	0.87	5.58e	7.01	3.28	6.94	6.02	3.49	1.13b	0.11	7.28
Estatio	6.42	7.33	6.66	6.61	5.68	3.87a	0.50	6.24b	5.32	3.11	6.84	5.07	2.98	0.69c	0.19	6.13
*F*-value	0.10	2.71	0.36	1.27	0.60	4.15	0.16	19.61	0.85	2.36	2.74	0.34	1.90	6.48	0.25	0.99
*p*	0.990	0.092	0.861	0.356	0.700	0.031	0.971	<0.0001	0.549	0.125	0.090	0.877	0.191	0.008	0.930	0.477
**S × R**																
*F*-value	0.79	2.05	0.46	1.38	0.92	1.90	1.65	6.97	1.49	2.11	0.27	0.37	5.40	4.51	0.45	1.82
*p*	0.527	0.178	0.718	0.310	0.470	0.199	0.245	0.010	0.283	0.169	0.844	0.777	0.021	0.034	0.724	0.213

*The means marked with different lowercase letters in row (for each factor) are significantly different (Tukey’s test, p ≤ 0.05). Identification of sensory attributes and scores: fruit appearance, external and internal appearance: shape, fruit shape; firm, fruit firmness by touch; fr col, color of the fruit; fl col, flesh color intensity; odor of the cut fruit: tom, tomato–like odor; green, green/grass odor; mouthfeel/texture attributes: thick, skin thickness; firm, flesh firmness; mea, flesh mealiness; juic, flesh juiciness; taste and after-taste: tom, tomato flavor; overall score, overall quality score.*

In experiment 2, the Clarabella scion yielded significantly higher scores for *fruit shape* and red color intensity of both *fruit skin* and *flesh.* Among the other sensory properties, differences were observed between scions for *sweet* taste and *tomato odor* features, and both were more intense in fruits from the Clarabella scion. Higher intensity *green* notes reminiscent of the grass, leaves and green fruit were recorded from the fruits of Estatio scion than from those of Clarabella scion cultivar. For the mouthfeel attributes, mealier flesh and more pronounced *tomato* flavor were determined in the fruits from Clarabella scion than from Estatio, which led to an overall better score of Clarabella in relation to Estatio (Table 2).

Rootstock affected the *green* odor attribute, *skin thickness* and *fruit sweetness* (Table 2). *Skin thickness* was the highest in fruits from plants where Clarabella was used as the rootstock and the lowest in fruits from plants grafted onto Maxifort rootstock, the latter of which did not differ from that of fruits from plants grafted onto Buffon rootstock.

Compared to the Buffon, Emperador and Maxifort rootstocks, the Estatio rootstock resulted in more intense *green* odor attributes. Moreover, compared with all the other rootstocks, the Estatio rootstock caused reduced pulp sweetness (Table 2).

**FIGURE 2:**
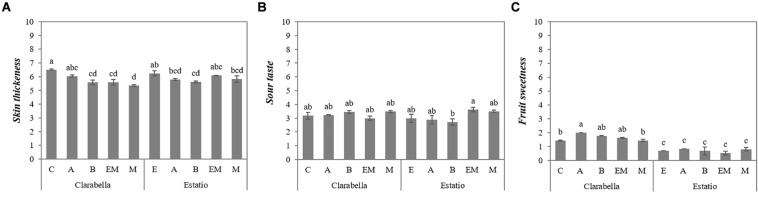
Scion × rootstock interaction effects on tomato fruit sensory attributes in the experiment 2. The results are expressed as the mean values ± SEs. The different lowercase letters indicate significant differences according to the Tukey test at *p* ≤ 0.05. Clarabella and Estatio are scions that were self-grafted or grafted onto Arnold (A), Buffon (B), Emperador (EM), or Maxifort (M) rootstocks. Identification: **(A)** Skin thickness; **(B)** Sour taste; **(C)** Fruit sweetness.

The interaction effects of scion × rootstock on the *skin thickness* and *sour* and *sweet* taste attributes are presented in Figure 2. The *skin thickness* of fruits from self-grafted Clarabella did not differ only from that of fruits from Clarabella grafted onto rootstock Arnold (Figure 2A). Thinner skin was observed for the fruits of Clarabella plants grafted onto other rootstocks (Buffon, Emperador and Maxifort) compared with fruits from self-grafted Clarabella plants. When Estatio was used as a scion, grafting onto only Buffon rootstock decreased the *skin thickness* compared with that of fruits from self-grafted Estatio plants (Figure 2A).

The intensity of the *sour* taste increased in fruits from plants in which Estatio was grafted onto Emperador compared with the Estatio grafted onto Buffon; however, there were no differences between the combinations in which Clarabella was used as a scion (Figure 2B).

Figure 2C shows that the *fruit sweetness* of fruits from Clarabella grafted onto Arnold, Buffon and Emperador rootstocks was higher than that of fruits from self-grafted Clarabella, Clarabella grafted onto Maxifort and all combinations in which Estatio was used as a scion.

### Volatile Profile Composition

#### Experiment 1

A total of 23 volatile compounds were identified in tomato fruit in experiment 1 (Table 3 and Supplementary Table 3). A higher concentration of total aldehydes, total alcohols and total volatile compounds was detected in the fruits of Clarabella scion compared to the fruits of Estatio scion.

**TABLE 3:** Effects of scions and rootstocks on volatile aroma compound concentrations in tomato fruits in the experiment 1.

Factor	Volatile compounds (μg/kg)												
	3MBal	Pal	Z3H + E2H	E2Hal	HEX	EE24Dal	TAl	3M2Bol	1P3ol	3MBol	nAol	1Hol	Z3Hol
**Scion (S)**													
Clarabella	82.32a	161.5a	129.1a	216.9	4.39	23.39	617.6a	8.27	16.13	24.37b	106.4a	134.1	4.17
Estatio	49.25b	111.6b	91.5b	206.9	4.81	17.02	481.1b	10.53	12.29	36.29a	65.3b	97.1	15.41
*F*-value	44.09	54.94	4.23	0.58	0.89	1.39	11.56	0.90	0.06	4.44	49.08	2.89	0.33
*p*	<0.0001	<0.0001	0.045	0.449	0.328	0.244	0.001	0.348	0.986	0.040	<0.0001	0.096	0.568
*F*-value	1.29	14.52	9.32	4.92	4.95	3.7	5.11	2.65	2.51	4.81	28.84	4.27	12.66
*p*	0.285	<0.0001	<0.0001	0.001	0.001	0.007	0.001	0.035	0.044	0.001	<0.0001	0,003	<0.0001
**R × S**													
*F*-value	1.00	1.04	4.77	15.82	0.53	1.98	4.26	5.48	3.34	16.18	0.72	0.51	0.41
*p*	0.376	0.362	0.013	<0.0001	0.594	0.151	0.020	0.007	0.045	<0.0001	0.493	0.604	0.663

	**PHEol**	**TAol**	**CAR**	**PHE**	**CPhy**	**HUM**	**CaRox**	**TTer**	**ßIon**	**EtBut**	**P + 1P3on**	**TVC**	

**Scion (S)**													
Clarabella	23.19b	316.6a	6.09b	35.66	5.94	7.87b	0.88b	56.40	1.15	1.47b	127.6a	1120.8 a	
Estatio	27.49a	264.5b	9.64a	31.95	5.35	12.61a	2.07a	61.62	0.93	4.64a	80.0b	892.8 b	
*F*-value	38.62	5.34	32.70	0.29	0.03	20.16	23.51	9.35	0.07	19.36	6.55	20.06	
*p*	<0.0001	0.002	<0.0001	0.594	0.859	<0.0001	<0.0001	0.054	0.789	<0.0001	0.014	<0.0001	
**Rootstock (R)**													
Clarabella	18.78c	358.4a	4.51c	43.85a	6.15	8.35ab	0.00c	62.85	1.49	1.35b	139.7a	1189.8 a	
Arnold	25.87b	256.5c	7.76b	28.76bc	6.13	13.17a	3.31a	59.15	1.08	5.42a	74.5b	845.7 c	
Buffon	24.23b	269.1bc	5.22c	37.65ab	5.49	9.09ab	1.06b	58.53	1.03	0.94b	135.6a	1137.3 ab	
Emperador	36.11a	337.2ab	9.71a	28.38c	6.59	11.03a	0.78b	56.49	1.32	1.09b	133.7a	1111.6 ab	
Maxifort	22.01b	291.9abc	8.43ab	37.31abc	5.19	9.25ab	1.24b	61.43	0.78	4.87a	91.5b	1055.5 b	
Estatio	14.93d	242.3c	7.49b	35.73abc	3.61	5.44b	0.73b	53.01	0.61	0.82b	83.1b	822.8 c	
*F*-value	52.68	3.87	6.02	3.42	1.80	3.59	16.59	2.10	1.33	7.45	3.81	7.97	
*p*	<0.0001	0.028	0.0003	0.011	0.132	0.008	<0.0001	0.090	0.271	<0.0001	0.006	<0.0001	
**R × S**													
*F*-value	67.13	3.22	34.77	1.94	2.52	12.00	37.36	15.22	1.64	10.85	6.01	8.05	
*p*	<0.0001	0.006	<0.0001	0.156	0.095	<0.0001	<0.0001	<0.0001	0.206	0.0002	0.005	0.001	

*The means marked with different lowercase letters in row (for each factor) are significantly different (Tukey’s test, p ≤ 0.05). Identification of volatile compounds, 3MBal, 3-methylbutanal; Pal, pentanal; Z3H + E2H, (Z)-3-hexenal + (E)-2-hexenal; E2Hal, (E)-2-heptanal; HEX, hexanal; EE24Dal, (E,E)-2,4-decadienal; TAl, total aldehydes; 3M2Bol, 3-methyl-2-butanol; 1P3ol, 1-peten-3-ol; 3MBol, 3-methylbutanol; nAol, n-amyl alcohol; 1Hol, 1-hexanol; Z3Hol, (Z)-3-hexen-1-ol; PHEol, phenylethanol; TAol, total alcohols; CAR, (+)-2-carene; PHE, (R)-(-)α-phellandrene; CPhy, (−)-(E)-caryophyllene; HUM, α-humulene; CaRox – (−)-caryophyllene oxide: TTer, total terpenes; βIon, β-ionon; EtBut, ethyl butyrate: P+1P3on, (+) α-pinen + 1-penten-3-one; TVC, total volatile compounds.*

The rootstock affected the concentration of volatile compounds in tomato fruits, except that of isovaleraldehyde, (−)-(*E*)-caryophyllene and β-ionone (Table 3). Clarabella as a rootstock yielded fruits with the highest concentration of (*Z*)-3-hexenal + (*E*)-2-hexenal. Compared with fruits from plants grafted onto Arnold and Estatio rootstocks, fruits from plants grafted onto Buffon, Emperador, and Clarabella rootstocks had higher (*E,E*)-2,4-decadienal concentrations. A higher concentration of 3-methyl-2-butanol was found in fruits from Emperador and Estatio rootstocks compared to the other tested rootstocks. Clarabella as a rootstock and Buffon rootstock yielded fruits with the lowest concentration of terpene (+)-2-carene. Furthermore, compared with Arnold and Emperador rootstocks, the Clarabella rootstock caused an increase in the concentration of (*R*)-(-)α-phellandrene (Table 3) in the fruits.

**FIGURE 3:**
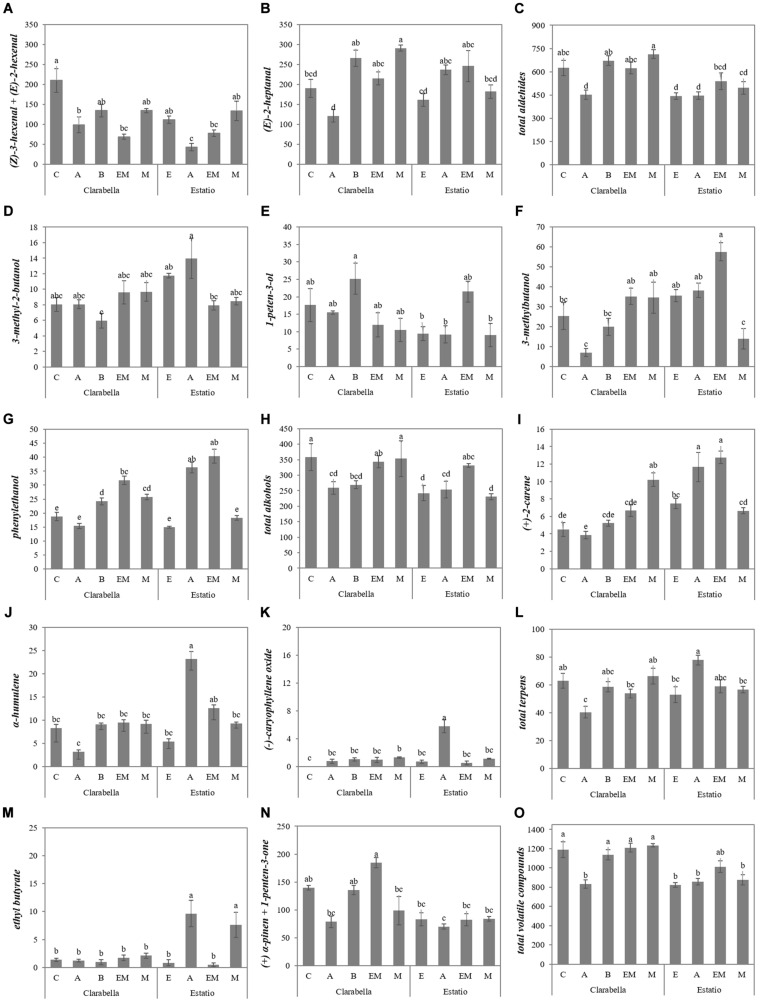
Scion × rootstock interaction effects on the concentrations of volatile aroma compounds in tomato fruits in the experiment 1. The results are expressed as the mean values ± SEs. The different lowercase letters indicate significant differences according to the Tukey test at *p* ≤ 0.05. Clarabella and Estatio are scions that were self-grafted or grafted onto Arnold (A), Emperador (EM), or Maxifort (M) rootstocks, while only Clarabella scions were grafted onto Buffon (B) rootstocks. Identification: **(A)** (Z)-3-hexenal + (E)-2-hexenal; **(B)** (E)-2-heptanal; **(C)** Total aldehides; **(D)** 3-methyl-2-butanol; **(E)** 1-peten-3-ol; **(F)** 3-methylbutanol; **(G)** phenylethanol; **(H)** Total alkohols; **(I)** (+)-2-carene; **(J)** α-humulene; **(K)** (-)-caryophyllene oxide; **(L)** Total terpens; **(M)** ethyl butyrate; **(N)** (+) α-pinen + 1-penten-3-one; **(O)** Total volatile compounds.

However, for the majority of identified volatile compounds, the main factors significantly interacted (Table 3, Figure 3, and Supplementary Table 3). Grafting Clarabella onto Maxifort increased the concentration of (*E*)-2-heptanal in the fruits compared with Clarabella grafted onto Arnold and self-grafted Clarabella, while when Estatio was used as scion, grafting did not induce a change in (*E*)-2-heptanal concentration among the examined rootstocks (Figure 3B).

Fruits from Clarabella plants grafted onto Emperador and Maxifort had higher concentrations of 3-methylbutanol than did fruits from Clarabella plants grafted onto Arnold (Figure 3F). While grafting Clarabella onto selected/tested rootstocks led to changes in volatile compound compositions, there were no differences between the combinations with Estatio as a scion for 1-penten-3-ol (Figure 3E), (+) α-pinen + 1-penten-3-one (Figure 3N), total aldehydes (Figure 3C) or total volatile compounds (Figure 3O). Likewise, compared with the other combinations, grafting of Clarabella plants onto Arnold decreased the total aldehyde (Figure 3C) and total volatile compound concentrations in the fruits (Figure 3O).

#### Experiment 2

The scion cultivar influenced 11 of 21 detected volatile compounds, total aldehydes, terpenes, and total volatile compound concentrations (Table 4). The scions in this experiment did not cause differences in concentrations of hexanal, 1-penten-3-ol, 1-hexanol, (*Z*)-3-hexen-1-ol, (−)-(*E*)-caryophyllene, (−)-caryophyllene oxide, ß-ionone or (+) α-pinen + 1-penten-3-one in the fruits (Table 4). The total aldehyde, total terpene and total volatile compound concentrations were higher in Clarabella scion fruits than in Estatio scion fruits.

**TABLE 4:** Effects of scions and rootstocks on volatile aroma compound concentrations in tomato fruits in the experiment 2.

Factor	Volatile compound (μg/kg)												
	3MBal	Pal	Z3H + E2H	E2Hal	HEX	EE24Dal	TAl	3M2Bol	1P3ol	3MBol	nAol	1Hol	Z3Hol
**Scion (S)**													
Clarabella	75.65a	179.2a	57.71b	525.7a	22.67	63.80a	924.7a	10.38b	37.78	27.10b	110.9b	34.46	43.56
Estatio	59.36b	114.1b	61.70a	270.2b	21.92	41.90b	569.2b	19.71a	31.89	38.27a	121.0a	32.25	44.01
*F*-value	6.55	142.6	27.88	100.1	0.24	16.80	102.2	19.64	0.45	37.57	8.3	0.08	1.08
*p*	0.021	<0.0001	<0.0001	<0.0001	0.632	0.0008	<0.0001	0.0004	0.510	<0.0001	0.011	0.791	0.315
**Rootstock (R)**													
Clarabella	64.08	156.4b	103.8a	491.7a	26.57	69.08a	911.6a	5.87b	44.85a	26.48c	126.6a	50.39a	54.19
Arnold	73.10	127.9c	64.9b	301.5c	25.31	40.81c	633.6c	18.58a	26.67cd	46.29a	100.3b	14.47d	29.04
Buffon	75.10	184.2a	47.0c	436.9ab	20.61	59.58ab	823.5ab	14.68a	37.64b	23.44c	126.2a	27.16c	39.38
Emperador	68.14	110.4cd	57.0bc	459.5a	19.88	59.51ab	774.6c	18.10a	39.82ab	36.72b	141.4a	33.79c	26.43
Maxifort	62.32	164.9b	49.4c	378.1b	22.64	45.91bc	723.4bc	16.81a	33.52bc	35.32b	99.9b	46.88b	62.17
Estatio	50.31	94.7d	58.3bc	238.9c	19.43	38.70c	500.5d	13.50a	25.11d	26.41c	104.1b	31.66c	49.11
*F*-value	0.97	21.4	27.4	3.3	0.57	2.34	3.54	3.57	9.13	10.35	11.5	37.84	2.44
*p*	0.462	<0.0001	<0.0001	0.029	0.721	0.089	0.020	0.023	0.0003	0.0001	<0.0001	<0.0001	0.080
**R × S**													
*F*-value	2.83	89.67	15.08	23.44	0.80	7.20	43.51	3.21	19.85	1.16	27.28	118.63	3.77
*p*	0.071	<0.0001	<0.0001	<0.0001	0.511	0.002	<0.0001	0.051	<0.0001	0.356	<0.0001	<0.0001	0.055

	**PHEol**	**TAol**	**CAR**	**PHE**	**CPhy**	**HUM**	**CaRox**	**TTer**	**ßIon**	**EtBut**	**P + 1P3on**	**TVC**	

**Scion (S)**													
Clarabella	19.19a	283.4	7.76b	11.44a	9.57	21.56a	5.10	55.44a	1.22	5.07a	125.7	1395.6 a	
Estatio	5.76b	292.9	10.58a	7.18b	9.12	11.94b	5.09	43.94b	1.03	2.22b	118.1	1027.5 b	
*F*-value	264.25	0.3	8.64	134.19	0.68	8.37	0.56	7.72	0.25	54.59	0.7	94.6	
*p*	<0.0001	0.324	0.010	<0.0001	0.422	0.011	0.466	0.013	0.624	<0.0001	0.392	<0.0001	
**Rootstock (R)**													
Clarabella	16.68a	335.1a	7.06b	10.42ab	7.44bc	24.58	5.68ab	55.20	0.86	4.74a	73.4d	1381.0 a	
Arnold	7.82c	243.2c	10.36a	11.26a	5.51d	11.96	6.68a	45.80	1.11	2.04cd	139.4ab	1065.3 c	
Buffon	15.44b	283.9abc	10.57a	7.21e	9.41b	16.71	4.16ab	48.07	1.14	4.08ab	170.6a	1331.4 a	
Emperador	11.80c	308.0ab	5.07b	9.86bc	18.11a	19.77	5.35ab	58.19	1.71	3.40bc	108.1bcd	1254.1 bc	
Maxifort	12.21bc	306.9ab	10.78a	8.33de	7.27bc	16.38	3.54b	46.32	1.12	4.84a	112.5bc	1195.2 b	
Estatio	7.24c	257.1b	11.10a	8.78cd	6.86bc	9.53	6.34a	42.63	0.39	1.46d	83.2cd	885.4 d	
*F*-value	5.79	8.8	8.59	15.20	9.72	0.94	2.90	0.74	2.04	3.69	8.1	7.2	
*p*	0.003	0.0007	0.0004	<0.0001	0.0002	0.482	0.047	0.606	0.127	0.021	0.0005	0.0001	
**R × S**													
*F*-value	7.46	31.54	6.77	3.70	3.03	0.88	3.78	0.47	1.90	46.67	4.98	79.07	
*p*	0.002	<0.0001	0.004	0.033	0.060	0.471	0.032	0.707	0.171	<0.0001	0.013	<0.0001	

*The means marked with different lowercase letters in row (for each factor) are significantly different (Tukey’s test, p ≤ 0.05). Identification of volatile compounds, 3MBal, 3-methylbutanal; Pal, pentanal; Z3H + E2H, (Z)-3-hexenal + (E)-2-hexenal; E2Hal, (E)-2-heptanal; HEX, hexanal; EE24Dal, (E,E)-2,4-decadienal; TAl, total aldehydes; 3M2Bol, 3-methyl-2-butanol; 1P3ol, 1-peten-3-ol; 3MBol, 3-methylbutanol; nAol, n-amyl alcohol; 1Hol, 1-hexanol; Z3Hol, (Z)-3-hexen-1-ol; PHEol, phenylethanol; TAol, total alcohols; CAR, (+)-2-carene; PHE, (R)-(−)α-phellandrene; CPhy, (−)-(E)-caryophyllene; HUM, α-humulene; CaRox, (−)-caryophyllene oxide: TTer, total terpenes; βIon, β-ionon; EtBut, ethyl butyrate: P + 1P3on, (+) α-pinen + 1-penten-3-one; TVC, total volatile compounds.*

The majority of the detected volatile compounds were affected by the rootstock cultivar (Table 4). The Clarabella as rootstock yielded fruits with twofold higher concentrations of (*Z*)-3-hexenal + (*E*)-2-hexenal. Higher concentrations of (*E*)-2-heptanal were detected in fruits from plants that had Clarabella and Emperador as a rootstock compared with the other rootstocks, except the plants grafted onto Buffon. However, Estatio rootstock and Arnold rootstock caused decreased contents of volatile compounds in the fruits. Arnold rootstock, followed by Clarabella as rootstock, yielded fruit with highest (*R*)-(−)α-phellandrene concentration.

No differences were noticed in the concentrations of 3-methylbutanal, hexenal, (*E,E*)-2,4-decadienal, (*Z*)-3-hexen-1-ol, alpha-humulene, or β-ionone among rootstock treatments (Table 4).

**FIGURE 4:**
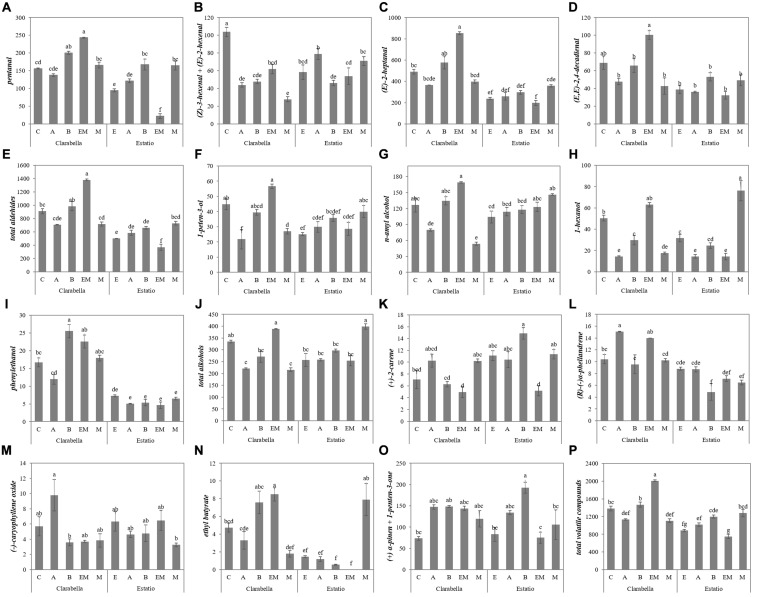
Scion × rootstock interaction effects on the concentrations of volatile aroma compounds in tomato fruits in the experiment 2. The results are expressed as the mean values ± SEs. The different lowercase letters indicate significant differences according to the Tukey test at *p* ≤ 0.05. Clarabella and Estatio are scions that were self-grafted or grafted onto Arnold (A), Buffon (B), Emperador (EM), or Maxifort (M) rootstocks. Identification: **(A)** pentanal; **(B)** (Z)-3-hexenal + (E)-2-hexenal; **(C)** (E)-2-heptanal; **(D)** (E,E)-2,4-decadienal; **(E)** Total aldehides; **(F)** 1-peten-3-ol; **(G)** n-amyl alcohol; **(H)** 1-hexanol; **(I)** phenylethanol; **(J)** Total alcohols; **(K)** (+)-2-carene; **(L)** (R)-(-)α-phellandrene; **(M)** (-)-caryophyllene oxide; **(N)** ethyl butyrate; **(O)** (+) α-pinen + 1-penten-3-one; **(P)** Total volatile compounds.

The variations in volatile compound composition caused by the scion × rootstock interaction effect are presented in Figure 4 and Supplementary Table 4. Between the 10 combinations, self-grafted Clarabella yielded the fruits with the highest concentration of (Z)-3-hexenal + (E)-2-hexenal (Figure 4B). Grafting of Clarabella plants onto Emperador increased the fruit concentration of 1-peten-3-ol and n-amyl alcohol compared to that in Clarabella grafted onto Arnold and Maxifort (Figures 4F,G). Fruits with the highest (*R*)-(−)α-phellandrene content were from Clarabella plants grafted onto Arnold, and this concentration did not differ from that of fruits of Clarabella plants grafted onto Emperador (Figure 4I). The lowest concentration of this volatile was detected in fruits from Estatio plants grafted onto Buffon, but the concentration did not differ from that of the fruits from Estatio grafted onto Emperador and Maxifort (Figure 4I).

## Discussion

Tomato fruit flavor is currently generally described as “classic tomato flavor” or “old-fashioned tomato flavor,” referring to the deterioration in the sensory quality of commercial tomato fruits. However, it is unclear how, why and whether fruit quality has truly changed.

The sensory quality of tomato fruit involves a large pool of key visual and organoleptic characteristics that are important from different standpoints in the tomato consumption chain. Nevertheless, sensory attributes assessed by quantitative descriptive sensory analyses have been poorly investigated in grafted tomato (Casals et al., 2018; Mauro et al., 2020).

The Clarabella and Estatio scions tested in the current study differed in *fruit shape* in both experiments (Tables 1, 2). However, the results were not uniform; in the first experiment, the fruits of Estatio were rounder and of more regular shape, while in the second experiment that was observed for Clarabella. Furthermore, compared with the other rootstocks, Clarabella as a rootstock led to irregular *fruit shape* (Experiment 1, Table 1), while in the second experiment, the effect of the rootstock on *fruit shape* was not recorded (Table 2). Commonly, the beef tomato type as observed in Clarabella is larger than the cluster tomato type as observed in Estatio. In the first experiment, we did not use bumblebees as pollinators (Yankit et al., 2018), which probably influenced the Clarabella scion yielding fruits of larger size. Regular fruit development in the second experiment when bumblebees were used conceivably caused Clarabella to manifest its genetic predispositions, resulting in generally rounder fruits. It was observed previously that grafting increases fruit indexes depending on scion (Turhan et al., 2011) and alters fruit height (Schwarz et al., 2013). Additionally, different rootstock-scion combinations can influence fruit mass (Khah et al., 2006; Kyriacou et al., 2017). However, in our study, where two scions and six rootstocks were tested, no significant scion × rootstock interaction effect for *fruit shape* was found (Tables 1, 2 and Supplementary Tables 1,2).

Texture-related attributes, which were assessed by touch and by physical sensations in the mouth, were affected by scion and rootstock (Tables 1, 2), and the interaction effect of scion × rootstock was significant for *flesh firmness* (Figure 1A) and *skin thickness* (Figure 2A).

Fruits of tomato plants where Estatio was used as the scion had lower *fruit firmness* than did those of plants in which Clarabella was used as the scion in experiment 1 (Table 1), which corroborates other reports of tomato fruit firmness diversity (Causse et al., 2010; Piombino et al., 2013). In both experiments, rootstock did not influence *fruit firmness* (Tables 1, 2), which is in accordance with the results of sensory evaluations on tomato fruits when the scion cultivar Sir Elyan was grafted onto three rootstocks (He-Man, Interpro and Armstrong) (Mauro et al., 2020). Nonetheless, evaluating more mature fruits obtained from the same rootstock-scion combinations led to changes in fruit firmness induced by the rootstock (Mauro et al., 2020). The mechanisms underlying fruit texture are complex and different molecular and biochemical mechanisms lead to changes in fruit firmness (Bertin and Génard, 2018). Reduction in intercellular adhesion, depolymerization and solubilization of cell wall components result in a decrease in firmness (Toivonen and Brummell, 2008), where the grafting impact could have a more pronounced effect. Overall, the grafting/rootstock impact on *fruit firmness* by mechanical method measurements show contradictory results: increases and no difference compared with those of self-grafted plants (Riga, 2015), reductions compared with those of self-grafted tomato (Schwarz et al., 2013), and no differences between non-grafted and grafted plants (Khah et al., 2006; Grieneisen et al., 2018). A recent meta-analysis study showed that 86% of the included data obtained from grafted tomato trials revealed no differences in *fruit firmness* (Grieneisen et al., 2018). Since *fruit firmness* is involved in fruit shelf-life and transport, preserving this sensory trait by grafting could be considered a positive outcome of the present study (Tables 1, 2).

*Skin thickness* is considered to be highly important for consumer acceptance, and relatively low scores are desirable (Causse et al., 2010). In the first experiment, in terms of *skin thickness*, only Clarabella differed from other rootstocks, while the variability of *skin thickness* among rootstocks was higher in the second experiment (Table 2). Clarabella as a rootstock yielded fruits with the highest *skin thickness* in both experiments, while the lowest *skin thickness* was perceived for fruits from Maxifort rootstock, though this parameter did not differ from that of Buffon (Table 2). This indicates the possibility of modifying this sensory attribute by selecting an appropriate rootstock. In the second experiment, the significant scion × rootstock interaction implied a different response of each scion grafted onto rootstocks in the study (Figure 2). Grafting both scions onto Buffon decreased the *skin thickness* of the fruits compared to that of self-grafted plants, whereas for Clarabella, the same effect was also found for fruits from plants grafted onto Emperador and Maxifort (Figure 2A). Casals et al. (2018) found that a decrease in skin perceptibility of fruits of plants under conventional management was the only positive effect of grafting among the sensory attributes they evaluated. However, pericarp thickness (cm) was not significantly influenced by grafting in soilless media or in soil (Qaryouti et al., 2007).

There is a lack of data on the effect of tomato grafting on *flesh mealiness*, as assessed by trained panels. In the literature, much attention has been given to understanding the basis and development of mealiness and many questions still need to be answered (Crisosto and Labavitch, 2002; Devaux et al., 2005; Toivonen and Brummell, 2008; Arefi et al., 2015). Fruit mealy texture is most likely caused by cell separation instead of cell rupture (Arefi et al., 2015), as indicated by the high correlation of mealiness and the number of fragments and cells produced (Devaux et al., 2005). Fruit mealy texture is negatively correlated with protein and uronic acid content and positively correlated with pectin content (Brownleader et al., 1999; Devaux et al., 2005). Additionally, as mentioned previously for *fruit firmness*, several mechanisms and a large amount of compositional remodeling are involved in texture-related sensory properties. In a review by Rouphael et al. (2018), rootstocks might influence fruit texture at the cell and tissue levels, which may be associated with the water and nutritional status of plants (Kyriacou et al., 2017). In our study, the Clarabella as rootstock yielded fruits with mealier flesh compared to those of other rootstocks, except Arnold (Table 1). Since *mealiness* is considered one of the most challenging attributes to determine, it has been suggested assess it in combination with *flesh firmness* and *flesh juiciness* (Barreiro et al., 1998). For the mouthfeel attribute *juiciness*, the effect of rootstock was not significant (Tables 1, 2), although the obtained data support an opposite relation with tomato fruit *juiciness* and *mealy perception* (Devaux et al., 2005). On the other hand, nearly the same effect of the commercial rootstock cultivars on *flesh firmness* as for *flesh mealiness* was noticed; the Clarabella as rootstock yielded the fruits with the firmest flesh (Table 1). The change in mealiness/juiciness can be potentially seen as quite positive, given that some consumers prefer one while other consumers prefer the other (Causse et al., 2010). In this way, the choice of rootstock and scion-rootstock combinations may influence the increase in diversification of tomato fruit texture-related attributes. In addition, based on the interaction effect, fruits from self-grafted Clarabella had firmer flesh than did those from Clarabella grafted onto Buffon and Maxifort, while differences in *flesh firmness* were not detected between fruits from grafted and self-grafted Estatio (Figure 1A).

The grafting/rootstock effect on taste attributes has been a topic of numerous studies [reviewed by Kyriacou et al. (2017) and Rouphael et al. (2010)]. In the present study, rootstocks induced changes in *fruit sweetness* in both experiments (Tables 1, 2), and in the first experiment, rootstocks additionally altered the *fruit sourness* intensity (Table 1). Tomato fruits from plants grafted onto Buffon were the sweetest (Table 1). With respect to *fruit sweetness*, an interaction effect between the scion with rootstock was observed in both experiments (Figures 1B, 2C). No differences in *fruit sweetness* were observed between fruits from Estatio grafted on different rootstocks (Figures 1B, 2C). However, in the first experiment, Clarabella grafted onto Buffon had sweeter fruits compared to fruits from self-grafted Clarabella (Figure 1B). Moreover, in experiment 2, compared with self-grafted Clarabella and Clarabella grafted onto Maxifort, Clarabella grafted onto Arnold resulted in increased *fruit sweetness* (Figure 2C). According to our observations, the Buffon and Maxifort rootstocks were less vigorous than were Clarabella, Arnold and Emperador, but there was no difference in the sweet taste of their fruits (Figure 1B). Thus, the results do not coincide with the outcomes of studies reporting a decrease in sugars when vigorous rootstocks that reduce assimilation flow to the fruits were used (Martínez-Ballesta et al., 2010). It is important to highlight that the results of sensory analyses conducted by panels can be only partially compared with the results of diverse chemical analyses (as was the case with the sugar content comparison above), since many other parameters lead to a real sensation in the mouth.

Product quality properties can be altered with the main intention to improve the quantity and the quality of compounds responsible for nutritive and/or sensory characteristics. Tomato flavor is the result of a diverse set of chemicals (such as sugars and acids) and, particularly, of volatile aroma compounds (Baldwin et al., 2000). Variation in volatile contents is exceptionally high within tomato cultivars (Tieman et al., 2012) and is further influenced by environmental and agronomic factors (Paolo et al., 2018).

In the present study, 23 volatile compounds were identified, including different aldehydes, alcohols, terpenes, ketones, and esters. Scions differed in their volatile compound profiles, and higher concentrations of total volatile compounds were detected in the fruits produced by Clarabella scions (Tables 3, 4). In this paper, we have shown that rootstocks exert a range of effects on tomato fruit volatile profiles (Tables 3, 4). In general, relatively low accumulation of total aldehydes, due to relatively low amounts of pentanal, (*E*)-2-heptanal and (*E,E*)-2,4-decadienal, was observed in fruits from plants grafted onto Arnold and Estatio. (*E*)-2-heptenal and (*E,E*)-2,4 decadienal are related to the sensory flavor attribute *tomato-like* (Krumbein et al., 2004), and links between the mentioned volatiles (Tables 3, 4) and *tomato-like* flavor were also confirmed in the present study (Tables 1, 2).

As a rootstock, Clarabella enhanced (*Z*)-3-hexenal + (*E*)-2-hexenal accumulation in the fruits (Tables 3, 4). (*Z*)-3-hexenal is among the most odor-active compounds and is described as *tomato/citrus* (Tandon et al., 2000) and *fresh green/sweet* (Krumbein and Auerswald, 1998), while (*E*)-2-hexenal is associated with the attributes *sweet* and *fruity* (Krumbein et al., 2004). Fruits from self-grafted Clarabella had higher (*Z*)-3-hexenal + (*E*)-2-hexenal concentrations than did fruits from Clarabella grafted onto Arnold and Emperador (Figure 3A). However, in the second experiment, grafting on all the commercial rootstocks reduced the fruit (*Z*)-3-hexenal + (*E*)-2-hexenal concentration compared to that from self-grafted Clarabella (Figure 4B). On the other hand, enhancement of (*E*)-2-heptanal was achieved by plants in which Clarabella was grafted onto Maxifort (Figure 3B) and Emperador (Figure 4C) compared to self-grafted Clarabella plants. In contrast, within the Estatio treatments, there was no difference in the concentrations of (*Z*)-3-hexenal + (*E*)-2-hexenal or (*E*)-2-heptanal between self-grafted plants and plants in which Estatio was grafted onto commercial rootstocks (Figures 3B, 4B,C). Thus, the results are partially in agreement with those of a study of grafting cocktail and conventional round truss tomato plants, where the majority of identified aldehydes did not differ between the fruits from self-grafted plants and plants grafted onto commercial rootstocks (Krumbein and Schwarz, 2013). Differences in responses to rootstocks are presumably due to barriers to metabolism as well as transmission between the rootstock and scion. It is known that previously mentioned volatile compounds are produced through the lipoxygenase (LOX) pathway by the sequential action of several enzymes involving unsaturated fatty acids as substrates (Yilmaz, 2001a). Although the mechanism by which grafting influences the synthesis of LOX-derived volatile compounds is still not clear, a recent transcriptome analysis indicated that physiological profiling and transcripts of aroma flavor-related genes notably changed in response to the different rootstocks, of which some transcripts could be upregulated, while the majority of the measured genes were expressed at lower levels in the self-grafted plants compared with the grafted plants (Zhao et al., 2018).

C_6_ compounds, the most abundant tomato fruit volatiles, show various contributions to tomato scents; however, using a metabolomics approach, Tieman et al. (2012) emphasized the contribution of (*Z*)-3-hexen-1-ol to flavor intensity, and Piombino et al. (2013) linked it to consumer acceptability. The (*Z*)-3-hexen-1-ol concentration was found to be rootstock-dependent in the study by Mauro et al. (2020), but Krumbein and Schwarz (2013) found no difference in (*Z*)-3-hexen-1-ol concentration between grafted plants and self-grafted plants. In the present study, the fruit concentration of (*Z*)-3-hexen-1-ol did not differ between the two scion cultivars, while in the first experiment, the highest concentration was observed in fruits from plants in which Estatio served as a rootstock (Tables 3, 4). Krumbein and Auerswald (1998) related (*Z*)-3-hexen-1-ol to *green* sensory attributes. In line with this, fruits with generally higher *green* notes (*grassy, herbal* notes) were recorded from plants having Estatio as a rootstock in both experiments (rootstock influence was non-significant in the first experiment; Tables 1, 2). In the second experiment, the intensity of the *green* sensory attribute of the fruits from plants having Estatio as a rootstock did not differ from that of fruits of plants in which Clarabella and Arnold served as a rootstock (Table 2).

Rootstock significantly affected the concentration of all identified alcohols in both experiments; however, no clear rootstock trend was observed (Tables 3, 4). On the other hand, for the majority of alcohols, the main factors significantly interacted in at least one experiment. For 1-penten-3-ol and phenylethanol, which have been described as having *pungent/butter* and *floral/sweet* odors and flavors, respectively (Verzera et al., 2011), a significant interaction was recorded in both experiments (Figures 3, 4). In the first experiment, among rootstocks grafted with Clarabella and rootstocks grafted with Estatio, there were no significant differences in 1-penten-3-ol concentration in the fruits (Figure 3E). Nonetheless, in the second experiment, among Clarabella combinations, the lowest concentration of 1-penten-3-ol was detected in fruits from Clarabella plants grafted onto Arnold, while compared with self-grafted Estatio plants, plants in which Estatio was grafted onto Maxifort presented increased fruit 1-penten-3-ol concentrations (Figure 4F). This result may be interesting in relation to consumer preferences for obtaining a less pungent tomato taste.

Many fruit and floral scents are the results of terpenoids (Pichersky et al., 2006), which are synthesized by parallel pathways in the cytosol (the mevalonate pathway) and in plastids (the methylerythritol 4-phosphate pathway) (Rohdich et al., 2003; Rohmer, 2003). Tomato fruits contain low concentrations of monoterpenes and sesquiterpenes (Petró-Turza, 1986; Buttery and Ling, 1993; Marković et al., 2007; Oluk et al., 2019), but due to their numerous roles in plants (they are involved in membrane structure, growth, signaling and defense mechanisms), breeding efforts are being made to increase their concentrations in tomato fruits (Davidovich-Rikanati et al., 2008). Studies on the grafting impact on terpenoids have seldom been performed. From the results presented in Tables 3, 4, it can be seen that rootstocks have similar potential with respect to the accumulation of fruit total terpenes. On the other hand, most of the single compound concentrations were rootstock dependent (the exceptions were (*−*)-(*E*)-caryophyllene and α-humulene in the first and second experiments, respectively), but there was no clear trend (Tables 3, 4). The interaction of scion x rootstock was significant for four out of the five identified terpenoids in the two conducted experiments (Figures 3, 4). Higher (+)-2-carene and (−)-caryophyllene oxide concentrations were observed in plants in which Clarabella was grafted onto Maxifort and plants in which Estatio was grafted onto Arnold compared to self-grafted plants (Figures 3I,K). The combination in which Estatio was grafted onto Arnold also led to increased fruit concentrations of α-humulene (Figure 3J). In the second experiment, grafting of Estatio onto Emperador induced a decrease in (+)-2-carene in the fruits, and grafting of Estatio onto Buffon decreased (R)-(−)α-phellandrene (Figures 4K,L). In terms of fruit sensory perception, terpenoids are associated with fresh *citrus-like* flavors, with warm, *peppery notes* of the tomato stem, which complements the aroma and attracts consumer attention (Berna et al., 2005). Several terpenoid concentrations could be enhanced under biotic and abiotic stress (Maes and Debergh, 2003), which is what the graft technique *per se* is (Riga, 2015). According to our data, it is clear that not all graft combinations cause the same response in terms of the accumulation of particular terpenoids; the rootstocks used obviously have different abilities to affect the concentrations of these compounds in the scions (Figures 3, 4).

Among the most common tomato volatiles is the apocarotenoid β-ionone, which is derived from enzymatic cleavage of carotenoids (Krumbein et al., 2004). It is linked to *violet-like* (Krumbein and Schwarz, 2013), *ripe tomato* (Buttery and Ling, 1993), and *ripe aroma* (Maul et al., 1998) scents and is positively correlated with *flavor*, *odor perception* and *fruit sweetness* (Mauro et al., 2020). Tieman et al. (2012) confirmed the contribution of apocarotenoid volatiles (geranial, 6-methyl-5-hepten-2-one, and β-ionone) to sweetness. The presence of β-ionone in the fruit is due to the decomposition of carotenoids, which are compounds responsible for fruit color (Kazeniac and Hall, 1970; Shi and Le Maguer, 2000; Tieman et al., 2006). In both experiments, we found no effect of scion, rootstock or their interactions on β-ionone concentrations (Tables 3, 4). This was confirmed by the results of the sensory analyses, where no effect of grafting on the *color of the fruit* or *flesh color intensity* was observed (Tables 1, 2).

In conclusion, the highest concentration of total volatile compounds was detected in tomato fruits from plants where Clarabella was used as rootstock, although this concentration did not differ from that in fruits of plants grafted onto Buffon or Emperador (Table 3). Similarly, in the second experiment, the highest fruit total volatile concentration was confirmed in fruits from scions grafted onto Clarabella and Buffon rootstocks (Table 4). Moreover, the results indicate a genotypic-dependent response, whereas in the first experiment, only plants in which Clarabella was grafted onto Arnold presented a decrease in fruit total volatile concentration compared that of self-grafted plants (Figure 3O). In the second experiment, differences among the tested combinations were greater, where Clarabella grafted onto Emperador stood out in terms of total volatile compound accumulation, followed by self-grafted Clarabella and Clarabella grafted onto Buffon (Figure 4P).

Among the combinations where Estatio was used as a scion, grafting onto Buffon and Maxifort induced an increase in total volatile compounds in the fruits, while compared with self-grafted Estatio plants, plants involving combinations of Arnold and Emperador did not present a decrease (Figure 4P).

Quantitative descriptive sensory analyses unite odor, flavor, retro-nasal attributes and senses that are exhibited during chewing and thus provide insight for elucidation of the high complexity of perception of tomato texture, flavor and aroma. As such, compared with fruits from self-grafted Clarabella plants, only fruits from Clarabella grafted onto Buffon had a better overall quality score (Figure 1C). Changes in fruit sensory traits induced by the rootstocks, scions or scion-rootstock combinations in this study show possibilities of increasing the variability of specific organoleptic properties of tomato fruits. Our data reveal that rootstocks did not negatively affect the overall quality score.

The differences that were observed among the studied properties between the experiments could be partially explained by the fact that the experiments were set up in production-like conditions. The influence of growing conditions on the results of this study also cannot be excluded, knowing that both volatile compounds and sensory properties are highly dependent on external climatic conditions.

These results could contribute to the knowledge about the effect of grafting on fruit volatile composition and sensory attributes, since, generally, the role of grafting in changes in fruit quality is poorly understood.

## Conclusion

It was demonstrated that modulation of the volatile compound composition and sensory profile could be achieved by using different rootstocks, scions and scion-rootstock combinations, and thus, tomato plants of these different combinations could be directed to a particular market as well as for the consumers’ desires and preferences. We have demonstrated that volatile compounds and sensory profiles were altered by the use of different rootstocks, scions and scion-rootstock combinations, which opens the possibility of testing particular combinations to fulfill consumer preferences in specific markets.

## Data Availability Statement

The original contributions generated for this study are included in the article/Supplementary Material, further inquiries can be directed to the corresponding author.

## Author Contributions

KŽ, GD, and SGB conceived and designed the study. GD, MM, GVS, MJŠ, and KŽ conducted the experiments. KBB conducted the sensory analyses. BS and ILJ analyzed the volatile aroma compounds. SGB and MJŠ analyzed the data. MJŠ drafted the manuscript. All authors read and approved the final manuscript.

## Conflict of Interest

The authors declare that the research was conducted in the absence of any commercial or financial relationships that could be construed as a potential conflict of interest.
