# Supplementary material for: Modification of the Sensory Profile and Volatile Aroma Compounds of Tomato Fruits by the Scion × Rootstock Interactive Effect
Source: Front Plant Sci. 2021 Jan 20;11:616431. doi: 10.3389/fpls.2020.616431 (PMC7855179; doi:10.3389/fpls.2020.616431)
Supplement: Supplementary file 1 [file Data_Sheet_1.PDF]

SUPPLEMENTARY MATERIAL

Figure S1

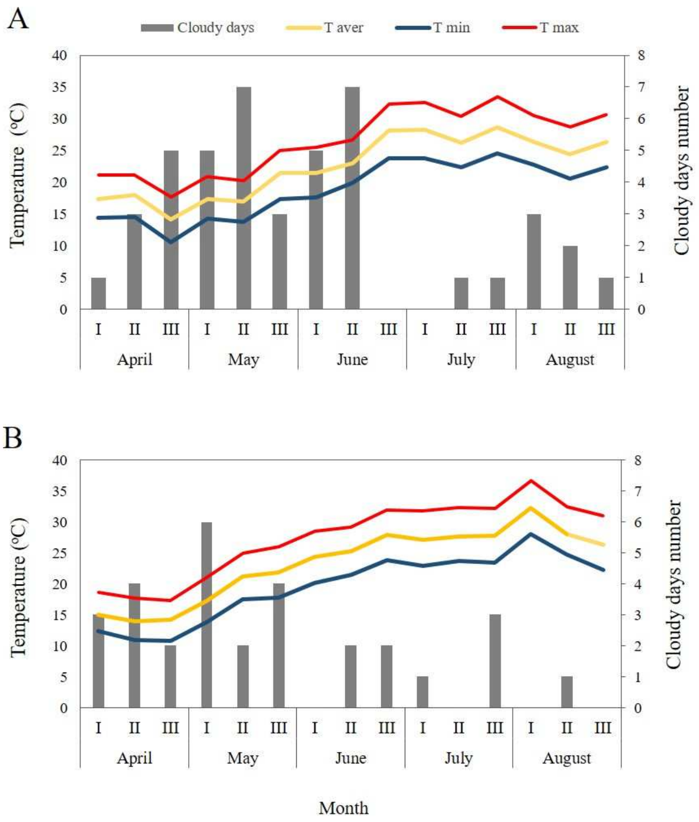

Figure S1. The values of temperature parameters and number of cloudy days registered in year 2016 A) and 2017 B)

Table S1. The tomato fruit sensory attribute intensities (scion × rootstock) in the spring-summer season of 2016.

	Sensory attributes and scores													
	Fruit appearance				Odour of the cut fruit			Mouthfeel/texture			Taste and after-taste		
	shape	*firm*	*fr col*	*fl col*	*tom*	*green*	*fru*	*thick*	*mea*	*juic*	*tom*	*sour*	*bitter*
Clarabella													
Clarabella	6.49	7.40	7.19	5.47	7.40	3.53	0.99	8.00	3.01	6.40	7.26	4.28	1.14
Arnold	7.32	7.42	7.18	5.98	7.44	3.59	1.09	7.41	3.23	6.97	7.22	3.70	1.04
Buffon	7.74	7.07	7.44	5.77	7.67	3.72	1.18	6.83	2.69	7.48	7.66	3.35	1.42
Emperador	7.47	7.85	7.74	6.25	7.62	3.82	1.14	7.38	2.80	7.20	7.49	3.51	0.79
Maxifort	7.26	7.04	7.15	6.28	7.75	3.53	1.25	6.74	2.68	7.31	7.65	3.30	0.69
Estatio													
Estatio	8.02	6.17	7.27	6.24	7.09	3.81	1.58	6.93	2.65	7.43	7.07	4.39	1.07
Arnold	8.22	6.31	7.58	6.06	7.14	3.58	1.53	7.00	2.35	7.28	7.06	4.25	0.82
Emperador	7.99	6.16	7.45	6.77	6.86	3.43	1.38	6.84	2.43	7.20	6.83	3.97	0.92
Maxifort	8.10	5.98	7.69	6.44	6.79	3.38	1.42	7.21	2.26	7.17	6.85	3.88	0.91

Identification of sensory attributes and scores: Fruit appearance - External and internal appearance: shape – fruit shape. firm – fruit firmness by touch. fr col – color of the fruit. fl col – flesh color intensity; Odor of the cut fruit: tom – tomato–like odor. green – green/grass odor; Mouthfeel/texture attributes: thick - skin thickness. mea – flesh mealiness. juic – flesh juiciness; Taste and after-taste: tom – tomato flavor.

Table S2. The tomato fruit sensory attribute intensities (scion × rootstock) in the spring-summer season of 2017.

	Sensory attributes and scores													
	Fruit appearance				Odour of the cut fruit			Mouthfeel/texture			Taste and after-taste		Overall score
	shape	*firm*	*fr col*	*fl col*	*tom*	*green*	*fru*	*firm*	*mea*	*juic*	*tom*	*bitter*	
Clarabella													
Clarabella	7.23	7.33	7.26	6.79	7.09	3.71	1.17	5.16	4.51	6.29	6.41	0.10	7.56
Arnold	6.89	7.31	7.26	6.99	7.24	3.32	1.26	4.96	3.84	6.88	6.67	0.16	8.19
Buffon	7.08	7.04	7.36	7.43	7.25	3.08	1.19	5.11	3.41	7.28	6.56	0.06	8.19
Emperador	7.32	7.15	7.20	6.90	7.24	3.02	1.28	5.22	4.01	6.99	6.63	0.07	8.16
Maxifort	7.00	6.77	7.16	6.79	7.17	2.83	1.11	4.90	3.53	7.02	6.71	0.12	7.78
Estatio													
Estatio	6.43	7.33	6.66	6.61	5.68	3.88	0.50	5.32	3.11	6.84	5.07	0.19	6.13
Arnold	6.69	7.13	6.34	6.14	4.98	3.61	0.35	5.53	2.94	6.67	4.92	0.04	5.91
Buffon	6.56	6.89	6.79	6.36	5.41	3.18	0.54	5.19	3.66	6.79	5.34	0.14	6.47
Emperador	6.31	7.44	6.53	6.29	5.56	3.63	0.31	5.47	3.31	6.76	5.19	0.08	6.44
Maxifort	6.73	7.12	6.76	6.48	5.74	3.64	0.64	9.13	3.03	6.87	5.33	0.09	6.78

Identification of sensory attributes and scores: Fruit appearance - External and internal appearance: shape – fruit shape. firm – fruit firmness by touch. fr col – color of the fruit. fl col – flesh color intensity; Odor of the cut fruit: tom – tomato–like odor. green – green/grass odor; Mouthfeel/texture attributes: thick - skin thickness. firm - flesh firmness. mea – flesh mealiness. juic – flesh juiciness; Taste and after-taste: tom – tomato flavor; Overall score – overall quality score.

Table S3. Volatile aroma compound concentrations (scion × rootstock) in tomato fruits in the spring-summer season of 2016.

	Volatile componud (mg/kg)									
	3MBal	Pal	HEX	EE24Dal	nAol	1Hol	Z3Hol	PHE	CPhy	ßIon
Clarabella										
Clarabella	64.7	133.8	4.27	23.01	105.4	183.3	0.58	43.9	6.15	1.49
Arnold	81.4	131.8	5.34	13.10	73.2	138.7	1.52	25.7	6.94	1.05
Buffon	77.0	161.6	3.85	29.07	108.7	79.8	5.46	37.6	5.50	1.03
Emperador	81.6	223.3	4.83	30.49	153.2	94.3	7.04	29.8	6.96	1.65
Maxifort	106.4	157.1	3.68	21.26	91.7	174.4	6.84	41.3	4.15	0.56
Estatio										
Estatio	49.7	103.6	4.28	12.21	47.1	80.5	13.00	35.7	3.61	0.61
Arnold	50.2	91.6	6.22	18.44	52.8	99.8	3.56	31.8	5.33	1.13
Maxifort	47.1	112.6	3.77	16.33	55.9	116.1	8.77	26.9	6.22	0.98
Emperador	50.0	138.7	4.95	21.04	105.7	92.2	6.29	33.3	6.24	1.00

Identification of volatile compounds. 3MBal - 3-methylbutanal; Pal – pentanal; HEX – hexanal; EE24Dal - (*E.E*)-2.4-decadienal; nAol - n-amyl alcohol; 1Hol - 1-hexanol; Z3Hol - (*Z*)-3-hexen-1-ol; PHE - (*R*)-(-)α-phellandrene; CPhy - (-)-(*E*)-caryophyllene; ßIon - ß-ionon.

Table S4. Volatile aroma compound concentrations (scion × rootstock) in tomato fruits in the spring-summer season of 2017.

	Volatile componud (mg/kg)								
	3MBal	HEX	3M2Bol	3MBol	Z3Hol	HUM	CaRox	*Tter*	ßIon
Clarabella									
Clarabella	64.0	26.6	5.87	26.5	64.2	24.6	5.69	55.2	0.86
Arnold	83.8	27.1	13.02	36.1	43.8	14.1	9.78	54.1	0.82
Buffon	75.7	20.2	8.37	13.5	20.0	18.5	3.58	50.7	1.49
Emperador	101.0	22.2	19.15	33.6	24.6	28.5	3.69	69.2	2.20
Maxifort	64.7	18.6	9.29	31.0	59.0	21.9	3.83	52.2	0.94
Estatio									
Estatio	50.3	19.4	13.51	26.4	49.1	9.5	6.35	42.6	0.39
Arnold	65.9	24.1	22.29	53.1	19.2	10.5	4.62	40.3	1.31
Buffon	74.4	21.1	21.00	33.4	58.8	14.9	4.76	45.5	0.78
Maxifort	59.9	26.6	24.35	39.6	65.3	10.8	3.26	40.4	1.30
Emperador	46.1	18.3	17.41	38.8	27.6	13.9	6.47	50.9	1.38

Identification of volatile compounds. 3MBal - 3-methylbutanal; HEX – hexanal; 3M2Bol - 3-methyl-2-butanol; 3MBol - 3-methylbutanol; Z3Hol - (*Z*)-3-hexen-1-ol; HUM - α-humulene; CaRox - (-)-caryophyllene oxide: TTer - *total terpens;* ßIon - ß-ionon.
